# Unveiling the Genetic Tapestry: Exploring *Rhizoctonia solani* AG-3 Anastomosis Groups in Potato Crops across Borders

**DOI:** 10.3390/plants13050715

**Published:** 2024-03-03

**Authors:** Syed Atif Hasan Naqvi, Aqleem Abbas, Muhammad Farhan, Rafia Kiran, Zeshan Hassan, Yasir Mehmood, Amjad Ali, Niaz Ahmed, Muhammad Zeeshan Hassan, Abdulwahed Fahad Alrefaei, Fatih Ölmez, Seung-Hwan Yang, Faheem Shehzad Baloch

**Affiliations:** 1 Department of Plant Pathology, Bahauddin Zakariya University, Multan 60800, Pakistan; atifnaqvi@bzu.edu.pk (S.A.H.N.); farhanbaloch6868@gmail.com (M.F.); rafaanikhan6868@gmail.com (R.K.); yasirmehmood@bzu.edu.pk (Y.M.); ranazeeshanhassan824@gmail.com (M.Z.H.); 2 Department of Agriculture and Food Technology, Karakoram International University, Gilgit Baltistan, Gilgit 15100, Pakistan; aqlpath@gmail.com; 3 College of Agriculture, University of Layyah, Layyah 31200, Pakistan; zeshan.hassan@ul.edu.pk; 4 Faculty of Agricultural Sciences and Technologies, Sivas University of Science and Technology, Sivas 58140, Türkiye; amjadbzu11@gmail.com (A.A.); fatih.olmez@sivas.edu.tr (F.Ö.); 5 Department of Soil Science, Bahauddin Zakariya University, Multan 60800, Pakistan; niaz.ahmad@bzu.edu.pk; 6 Department of Zoology, College of Science, King Saud University, P.O. Box 2455, Riyadh 11451, Saudi Arabia; afrefaei@ksu.edu.sa; 7 Department of Biotechnology, Chonnam National University, Yeosu 59626, Republic of Korea; 8 Department of Biotechnology, Faculty of Science, Mersin University, Yenişehir, Mersin 33343, Türkiye

**Keywords:** evolutionary genetics, *Solanum tuberosum*, hyphal anastomosis reactions, heterokaryosis, phylogenetic analysis

## Abstract

The current study was carried out to screen 10 isolates (ARS-01–ARS-10) of *Rhizoctonia. solani* from potato tubers cv. Kuroda, which were collected from various potato fields in Multan, Pakistan. The isolates were found to be morphologically identical, as the hyphae exhibit the production of branches at right angles and acute angles often accompanied by septum near the emerging branches. Anastomosis grouping showed that these isolates belonged to AG-3. A pathogenicity test was performed against the susceptible Kuroda variety and among the isolates, ARS-05 exhibited the highest mean severity score of approximately 5.43, followed by ARS-09, which showed a mean severity score of about 3.67, indicating a moderate level of severity. On the lower end of the severity scale, isolates ARS-06 and ARS-07 displayed mean severity scores of approximately 0.53 and 0.57, respectively, suggesting minimal symptom severity. These mean severity scores offer insights into the varying degrees of symptom expression among the different isolates of *R. solani* under examination. PCoA indicates that the severe isolate causing black scurf on the Kuroda variety was AG-3. A comprehensive analysis of the distribution, genetic variability, and phylogenetic relationships of *R. solani* anastomosis groups (AGs) related to potato crops across diverse geographic regions was also performed to examine AG prevalence in various countries. AG-3 was identified as the most widespread group, prevalent in Sweden, China, and the USA. AG-5 showed prominence in Sweden and the USA, while AG-2-1 exhibited prevalence in China and Japan. The phylogenetic analysis unveiled two different clades: Clade I comprising AG-3 and Clade II encompassing AG-2, AG-4, and AG-5, further subdivided into three subclades. Although AGs clustered together regardless of origin, their genetic diversity revealed complex evolutionary patterns. The findings pave the way for region-specific disease management strategies to combat *R. solani’s* impact on potato crops.

## 1. Introduction

The potato (*Solanum tuberosum*) stands as the primary food crop among tuber-producing plants and ranks as the fourth most significant staple globally, following rice, wheat, and maize. The production of protein, minerals, and dry matter per unit area is higher in potatoes than in cereal crops [1]. Potatoes are cultivated extensively in 130 countries, with a global output value of USD 63.6 billion in 2016 [2]. The world’s total production of potatoes in the year 2021 was 0.37611997439 billion tons from the total cultivated area of 18,132,694 ha [3]. *Rhizoctonia solani* Kuhn (teleomorph, “*Thanatephorus cucumeris* (Frank) Donk”) is a soil-borne fungus responsible for economically significant potato diseases. The *Rhizoctonia* genus contains a wide variety of filamentous fungi owing to its anamorphic, infertile condition and absence of sexual spores (teleomorphic stage). Several commercially and geographically significant plant diseases, such as those caused by *Rhizoctonia solani*, are attributed to members of this genus of fungus [4]. Julius Kuhn, in 1858, observed *R. solani* on potatoes, which is soil-borne in nature, with a destructive lifestyle, complex biology, and worldwide distribution. It is found naturally as hyphae and sclerotia and may infect a broad variety of hosts. Sclerotia are rigid structures, hyphal clumps that protect the fungus *R. solani* during unfavorable environmental conditions. *R. solani* spread mainly by sclerotia on the contaminated tuber, plant debris, by wind, water, or other cultural practices such as seed transportation, and tillage [5]. Anastomosis groups (AGs) associated with potato pathogens are typically pathogenic. AGs are a classification system used for grouping fungi, particularly those belonging to the genus *Rhizoctonia*, which includes several plant pathogens. In the context of potato cultivation, certain anastomosis groups (AGs) of *R. solani* are acknowledged as pathogenic, leading to the occurrence of diseases such as black scurf and stem canker in potatoes. These pathogenic AGs can adversely affect the growth and yield of potato crops. However, not all AGs within the genus *Rhizoctonia* are pathogenic to potatoes or other plants; some may have different host preferences or may not cause significant harm to potato crops. *R. solani* causes two types of symptoms in potatoes, viz., stem canker and black scurf [6,7]. Stem canker symptoms arise during the potato planting to harvest, and most serious damage occurs soon after planting. Plant vitality is compromised, plants are weakened, and either the number of stems or the number of stands is reduced as a result of the girdle, which is caused by cankers forming at the base of the stem and disrupting the usual upward and downward motions of water and carbohydrates [8]. Black scurf is the most easily observable symptom on the tuber as sclerotia. Dark brown to black-colored sclerotia is formed on tubers. Tubers become irregularly shaped, malformed, and cracked; consequently, their market value reduces [7]. Although several different *R. solani* AGs have been linked to potatoes, AG3-PT is the primary causal agent of black scurf and stem canker [8]. Variable pathogenicity and disease types have been linked to AG2-1, AG2-2, AG2-2IIIB, AG4, and AG5, whereby AG2-1 predominantly relates to stem canker, exhibiting diverse genetic and virulence features, while the three homogeneous groups (HGI, HGII, and HGIII) within AG4 primarily induce canker symptoms and, to a lesser extent, black scurf; additionally, AG1, AG7, AG8, and AG9 are among the other AGs occasionally documented to infect potatoes [7,9]. Different anastomosis groups (AGs) of *Rhizoctonia solani* have been associated with specific symptoms in potatoes, with AG8 primarily affecting the roots and AG7 spreading to the stalks, stolons, and tubers, whereas sporadic reports of AG1-1B, AG6, AG10, AG11, AG12, and AG13 on potatoes also exist, but the symptoms caused by many of these AGs remain unconfirmed [7].

*R. solani* may persist continuously in the soil or on the surface of potato tubers as sclerotia and potentially infect young potato plants during spring germination; although sclerotia production on tubers is possible at any time, it frequently occurs as the tubers mature, and *R. solani* develops disease on potato plants through three forms of inoculum: basidiospores, mycelium fragments, and sclerotia [10]. Basidiospores of *R. solani* are normally present on the surface of soil, leaves, and stems and transmitted by air, causing the disease to spread to aerial parts of plants [4]. The presence of mycelia fragments and sclerotia of *R. solani* in the soil or on potato tubers can lead to disease susceptibility in various plant parts, causing significant damage to the plant, however, studies conducted over the years have failed to definitively establish a single inoculum source’s causal involvement in disease onset [11]. Contrary findings from various studies have shown that among the sources of inoculum, seed negatively affects plant emergence, while tuber inoculum inhibits sprout emergence and causes girdling of sprouts, leading to stem canker; soil-borne inoculum has been associated with infection of stems and stolons, as well as the development of black scurf and stem canker, underscoring the significance of both inoculum sources in disease progression [10]. The severity of a disease is directly correlated with its inoculum load, whereby mycelia from a growing sclerotium of *R. solani* invade plants upon sensing molecular exudates such as amino acids, carbohydrates, organic acids, and phenols secreted by potato plants [12]. Within a few hours of the initial touch, the hypha compresses and develops in a specific orientation, initiating over the epidermal cells of the potato plants while T-shaped hyphal branches firmly adhere to the host skin, forming dense infection cushions, before actively penetrating the host [13]. Infection pegs, formed when the hyphal ends of infection cushions swell, pierce the cuticle and epidermal cell walls to gain access to the host’s epidermis and exterior layer of cortex [14].

Although disintegrating enzymes such as cutinases, pectinases, and xylanases are likely involved in infection and penetration, hydraulic pressure is utilized to establish initial contact [15]. Necrotic lesions on the epidermal tissue of branches, roots, and stolons, as well as damping-off of the young potato plants, are symptoms of the fungus’s destructive growth within the host [16]. Two disease complexes, stem canker and black scurf, are economically significant because they devastate the quality and quantity of potato crops all over the world [17]. *Rhizoctonia* causes an annual potato production loss ranging from 7% to 36% worldwide, with Western Siberia contributing to more than 50% of the total crop yield loss [18]. *R. solani* is hard to control because it is soil-borne in nature, and the fungus’s diverse host range adds to the difficulty [19]. The use of fungicides has been widespread in the control of plant diseases. However, their extensive use has led to serious environmental and health concerns. Therefore, there is a growing interest in the discovery and development of new and improved fungicides that are based on sustainable and environmentally friendly approaches [20]. Breeding-resistant potato cultivars are regarded as an environmentally safe and successful technique for *R. solani* management [21]. Understanding the genetic diversity of *R. solani* is crucial for the development of innovative management strategies, such as creating varieties resistant to *Rhizoctonia* [22,23,24]. The division of *R. solani* into anastomosis groups explains its genetic variability and, presently, 14 anastomosis groups have been identified (AG 1-13 and AG-BI), delineated based on hyphae fusion, shape, aggressiveness (pathogenicity), physical appearance, and DNA homogeneity [25,26,27]. Certain AGs are further classified based on the type of anastomosis, physiological and morphological features, pathological, biochemical, biomolecular, inherent, and DNA resemblance qualities [22]. For instance, AG-1 contains six subgroups, viz., IA, IB, IC, ID, IE, and IF, whereas AG-4 is divided into three subgroups, e.g., (HGI, HGII, and HGIII), while AG-2 is divided into nine (1, 2, 3, 4, t, Nt, 2IIIB, 2IV, and 2LP) subgroups [22]. Infection of potatoes with *R. solani* AG3-PT may cause typical disease signs such as stem canker and black scurf and various molecular indicators have been employed for detection and characterization, including techniques like ISSR (inter-simple sequence repeats), SSR (simple sequence repeats), SNPs (single nucleotide polymorphisms), AFLP (amplified fragment length polymorphisms), RFLP (restriction fragment length polymorphisms), RAPD markers derived from randomly amplified polymorphic DNA, EK (electrophoretic karyotyping), sequence analysis involving rDNA ITS1-5.8 S, and DNA-DNA hybridization [28].

Due to the presence of multiple recurrent tandem copies within the genetic content of all fungi, the examination of the rDNA ITS1-5.8 S-ITS2 region through sequence analysis has proven to be a superior method for investigating genetic diversity and the evolutionary connections within AG groups and AG subgroups of *R. solani* [22,29]. The verification of AG classification relies on conventional hyphal anastomosis responses, while the sequences of the rDNA ITS1-5.8S-ITS2 region evolve rapidly and are flanked by highly conserved nucleotide sequences, situated within the 18S and 5.8S rRNA genes (ITS1), as well as the 5.8S and 28S rRNA genes (ITS2) [22]. Genetic variability of *R. solani* causing potatoes black scurf and stem canker has been found in all areas of the world; however, there has been a dearth of effort to explore *R. solani* AG’s worldwide variation in genetics and geographical distribution related to potatoes [8]. Keeping in view the importance of *R. solani* AG’s genetic variation and pathogenic potential on potatoes, the present research aims to achieve the following objectives: (1) isolation, identification, and characterization of AG isolates from the different potato fields of Multan, Pakistan; (2) to identify the most commonly discussed and prevalent AG types related to potato; (3) to investigate the genetic makeup of AG types using rDNA ITS1-5.8S-ITS2 sequence examination, and (4) to establish the correlation between origins of geography and the genetic variability of AG types.

## 2. Results

### 2.1. Isolation, Identification and Pathogenicity of Rhizoctonia Solani Isolates

**Figure 1:**
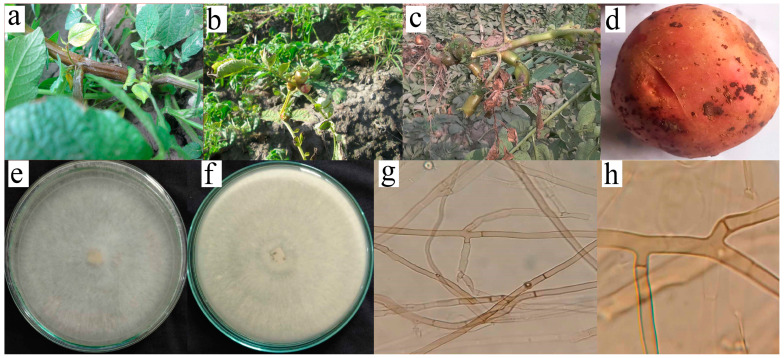
Symptomatology, morphological characteristics and pathogenicity of *R. solani* AG-3 ARS-05 isolate. (**a**) Whitish mold growth on foliage. (**b**) Rolling of leaves with aerial tubers. (**c**) Green-colored aerial tubers with stem canker. (**d**) Black sclerotia on tubers. (**e**) Growth of *R. solani* in Petri plate. (**f**) Growth of *R. solani* back side of Petri plate. (**g**,**h**) mycelium, right-angled branches having a constriction at the point of origin.

**Figure 2:**
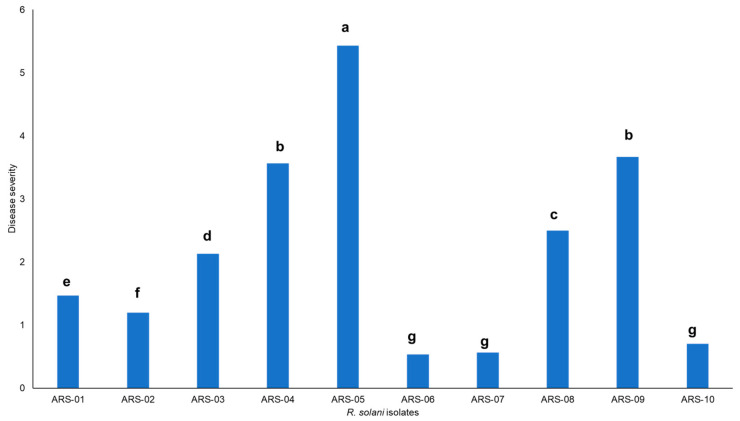
Pathogenicity test of ten isolates associated with black scurf caused by *R. solani.* Means followed by the same letters in each bar are not statistically different (*P < 0.05*).

Severe infection of *R. solani* was observed in the surveyed fields with characteristic symptoms, i.e., whitish grey mold growth on foliage, rolling of leaves with aerial tubers, and green-colored aerial tubers with stem canker (Figure 1). Cultures of the fungus were identified as *R. solani*, isolated, and characterized based on both morphological and microscopic features (Figure 1). During the initial stages of growth, the mycelia of all isolates displayed a light brown hue, accompanied by the prolific development of aerial hyphae throughout the entire growth cycle. With time, as the cultures matured, there was a noticeable darkening of their color, with the majority assuming a very dark brown appearance after 21 days. This morphological transition over time added a dynamic element to the fungal cultures’ characteristics, highlighting the evolution of their visual attributes as they progressed through different stages of development. Under microscopic examination, a lengthy mycelium was observed, typically becoming noticeable during the later stages of growth. However, in many cases, a lengthy mycelium tends to become more pronounced during the later stages of fungal growth, often beyond 10–14 days of incubation, though this can vary widely. The hyphae exhibit the production of branches at right angles and acute angles, often accompanied by a septum near the emerging branch (Figure 1). Further PCoA indicates severe isolate causing black scurf on Kuroda variety is AG-3. The assessment of mean severity scores for ten isolates of *R. solani* showed that among the isolates, ARS-05 exhibited the highest mean severity score of approximately 5.43, indicating a relatively more severe manifestation of symptoms. Following closely, ARS-09 had a mean severity score of about 3.67, indicating a moderate level of severity. On the lower end of the severity scale, isolates ARS-06 and ARS-07 displayed mean severity scores of approximately 0.53 and 0.57, respectively, suggesting minimal symptom severity. The remaining isolates fell within the spectrum of severity, with mean scores ranging from 1.2 to 3.57. These mean severity scores offer insights into the varying degrees of symptom expression among the different isolates of *R. solani* under examination (Figure 2).

### 2.2. Distribution of Anastomosis Groups

**Figure 3:**
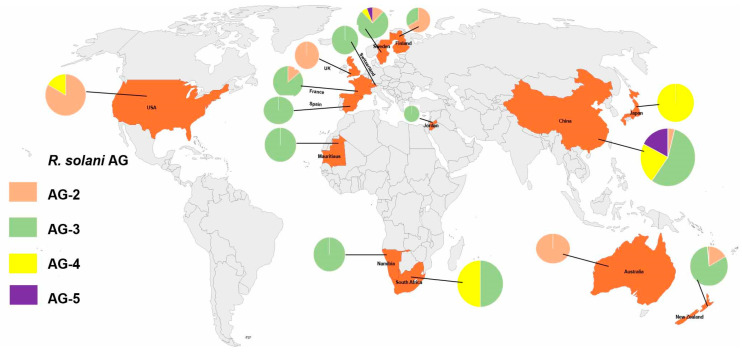
Global prevalence of *R. solani* anastomosis groups (AG) related to potatoes using GenBank sequences. The data in the pie charts represent the major AG found in each locality. The map illustrates the worldwide prevalence of distinct *R. solani* anastomosis groups (AG) concerning potato cultivation. The depicted regions showcase the predominant AG types observed in each locality, based on data extracted from GenBank sequences. The pie charts integrated into the map offer a concise representation of the proportional prevalence of specific AG isolates within each geographical area. These pie charts elucidate the prevalence percentages of various AG types, emphasizing their distribution patterns within the potato agricultural landscape. It is crucial to note that the primary purpose of this representation is to delineate the prevalence and distribution of different AGs associated with potato cultivation globally. The depiction of territories and boundaries solely serves to demarcate geographic regions for clarity in presenting the prevalence data and does not imply or endorse any geopolitical claims or disputes. The AG distribution data depicted in this figure elucidate the prevalence of distinct *R. solani* AGs across various regions, providing valuable insights into the global landscape of potato-associated *R. solani* strains. This representation aids in understanding the prevalence patterns of these AG types and their significance in potato cultivation on a global scale.

The distribution of anastomosis groups (AGs) in different countries depicted that the AGs reported up to now, AG-2 has been found in Sweden (2 occurrences), China (3 occurrences), the USA (5 occurrences), New Zealand (24 occurrences), Finland (2 occurrences), France (1 occurrence), the UK (3 occurrences), Australia (1 occurrence), and Spain (0 occurrences). AG-3 has been reported in Sweden (13 occurrences), Namibia (2 occurrences), Mauritius (1 occurrence), Switzerland (4 occurrences), China (42 occurrences), South Africa (3 occurrences), New Zealand (116 occurrences), Finland (1 occurrence), France (6 occurrences), Spain (2 occurrences), and Jordan (20 occurrences). AG-4 has been observed in Sweden (1 occurrence), China (17 occurrences), South Africa (3 occurrences), the USA (1 occurrence), Japan (1 occurrence), and New Zealand (1 occurrence). AG-5 has been present in Sweden (1 occurrence), China (13 occurrences), New Zealand (1 occurrence), and Australia (1 occurrence) (Figure 3).

### 2.3. Anastomosis Groups Based on Geographical Region: Number of Studies Reported

The findings of various studies regarding the distribution of anastomosis groups (AGs) in different geographical regions represent a specific AG, labeled from AG-2 to AG-5. The columns of the table indicate the number of studies conducted in specific countries or regions, such as Sweden, Namibia, Mauritius, Switzerland, China, South Africa, the USA, Japan, New Zealand, Australia, the UK, Finland, France, Spain, Switzerland, and Jordan, that reported the presence of the respective AG. Among the AGs, AG-3 showed the highest occurrence, being reported in 29 studies conducted in Sweden, 37 in China, and 24 in the USA. AG-5 was identified in 12 studies in Sweden and 9 in the USA. AG-4 HGII was reported in a single study in both Switzerland and China. AG-2-1, on the other hand, was prevalent in 24 studies in China and 37 studies in Japan. AG-3 PT was exclusively found in 24 studies conducted in Namibia. Several AGs, such as AG-4, AG-11, and AG-BI, showed limited or no occurrence across the studied geographical regions. Likewise, AG-2 was reported only in Sweden and Spain. Overall, AGs exhibited varying distributions across the surveyed countries, with AG-3, AG-5, AG-4 HGII, AG-2-1, and AG-3 PT being the most frequently observed. These findings provide valuable insights into the genetic relatedness of AGs in potatoes across different geographic locations and contribute to our understanding of potato pathogenicity and diversity (Table 1).

### 2.4. Genetic Diversity of Anastomosis Groups

**Figure 4:**
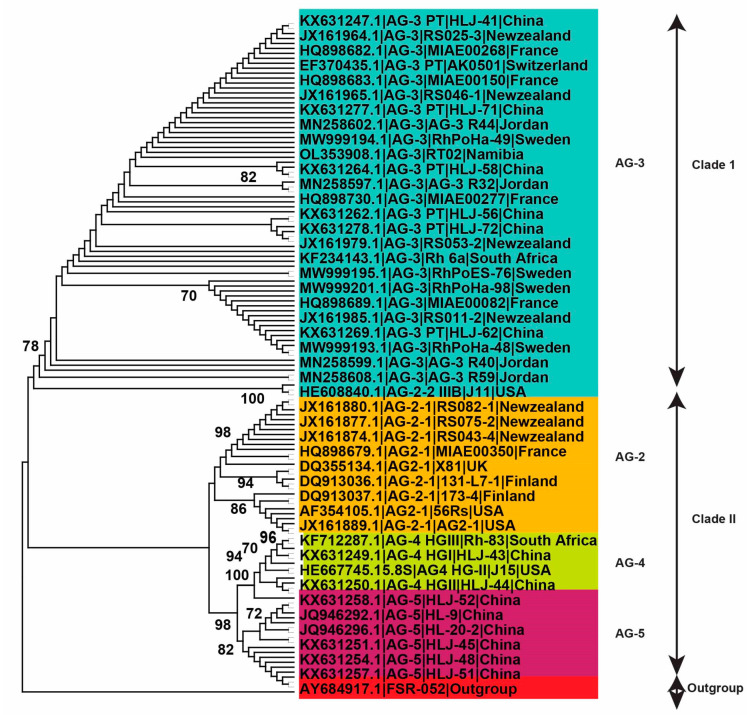
Genetic relatedness among potato AG (anastomosis groups) using a Maximum Likelihood (ML) analysis. The trees were built using accession numbers, isolate information, the geographic background, and unique AG designation. Various shades reflect different AG-associated clades and subclades. To ensure the robustness of the tree branches, thousands of replications were performed using bootstrap analysis. The values displayed at nodes indicate the bootstrap values, with only those greater than or equal to 70 being shown. To root the tree, the outgroup *Athelia rolfsii* FSR-052 (GenBank Accession No. AY684917) was used. The figure’s scale bar represents a genetic difference of 0.2 for horizontally oriented segment length. This information provides insights into the evolutionary relationships and genetic variations among potato AG.

**Figure 5:**
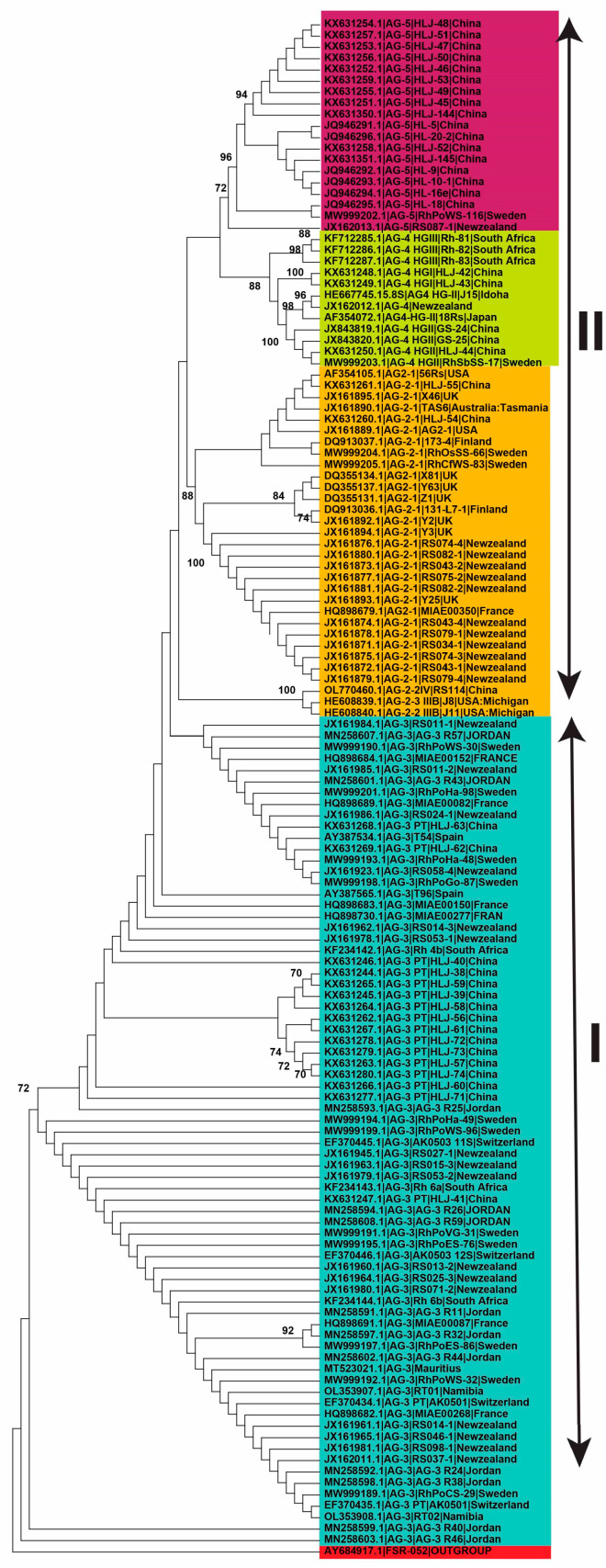
Genetic relatedness among potato AG (anastomosis groups) using a Maximum Likelihood (ML) analysis. The trees were built using accession numbers, isolate information, geographic background, and unique AG designation. Various shades reflect different AG-associated clades and subclades. To ensure the robustness of the tree branches, thousands of replications were performed using bootstrap analysis. The values displayed at nodes indicate the bootstrap values, with only those greater than or equal to 70 being shown. To root the tree, the outgroup *Athelia rolfsii* FSR-052 (GenBank Accession No. AY684917) was used. The figure’s scale bar represents a genetic difference of 0.2 for horizontally oriented segment length. This information provides insights into the evolutionary relationships and genetic variations among potato AG.

**Figure 6:**
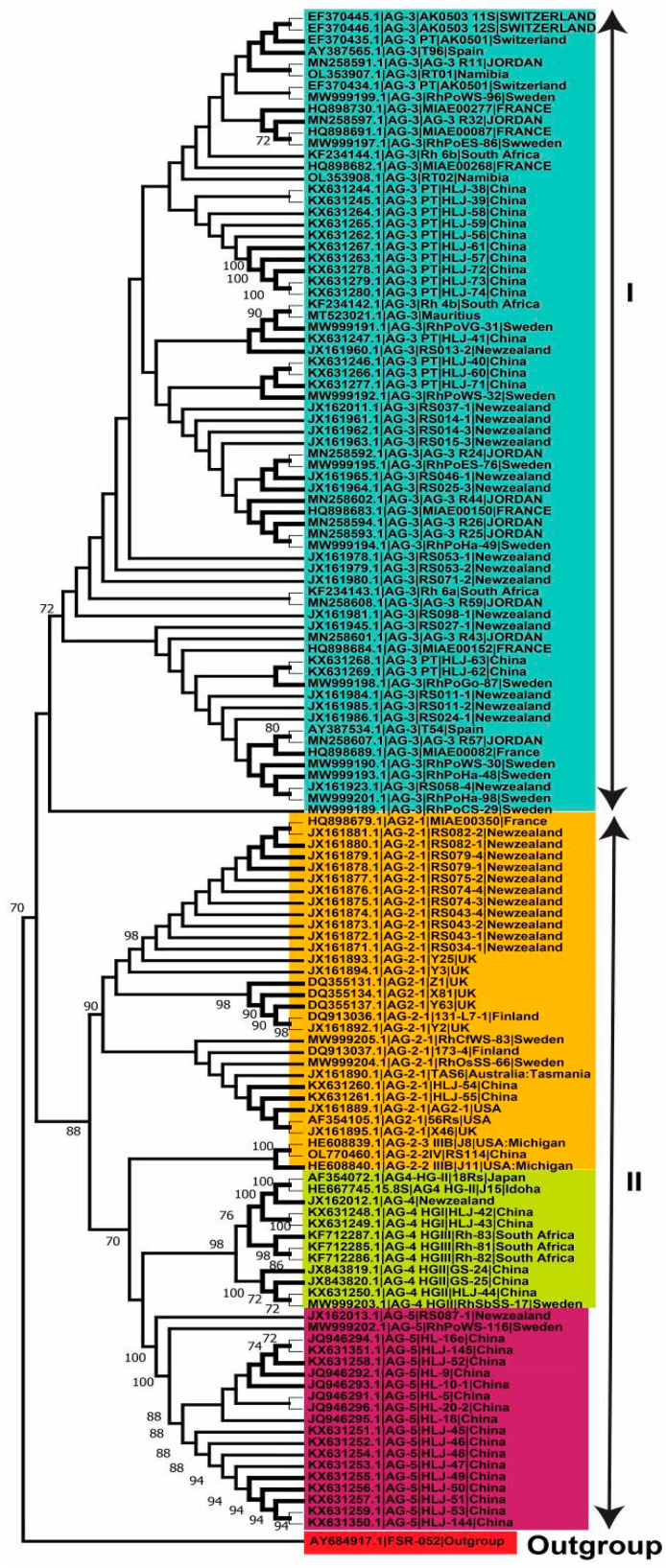
The genetic relatedness among potato AG (anastomosis groups) was examined through Neighbor-joining (NJ) analysis, which was employed to construct the trees. Each AG is represented by accession numbers, followed by information on the isolate, geographic location background, and AG classification. To establish the reliability of the tree branches, numerous replications (thousands) were performed using bootstrap analysis. The values displayed at nodes represent bootstrap values, with only those greater than or equal to 70 being shown. For tree roots, the outgroup *Athelia rolfsii* FSR-052 (GenBank Accession No. AY684917) was utilized. The scale bar in the figure corresponds to a genetic distance of 0.05, showing the length of a branch that runs horizontally. The use of Neighbor-joining analysis in studying potato AG’s genetic relatedness provides valuable insights for understanding their evolutionary patterns and relationships.

In the present investigation, 290 *R. solani* AG linked with potatoes had first been gathered from the scientific papers, and a big phylogenetic tree was constructed (Figure 4). However, only 136 AG from recognized isolates with known isolate names and geographical locations were utilized to investigate genetic variation and lineage (Figure 4). Using the NJ, ML, and MP approaches, analysis of sequences classified AG into two primary clades. The 136 AG isolates were divided into two separate clades, with Clade I consisting of AG-3 isolates and Clade II consisting of AG-2, AG-4, and AG-5 isolates. Clade II was further clustered into three distinct subclades, with sufficient bootstrap support for each subclade. AG-2 subclades include isolates of subgroups AG-2-2 and AG2-1, whereas the other two subclades contain isolates of AG-4 and AG-5 (Figure 4, Figure 5 and Figure 6).

### 2.5. Genetic Relatedness among and within Clades and Subclades Representing Anastomosis Groups

**Figure 7:**
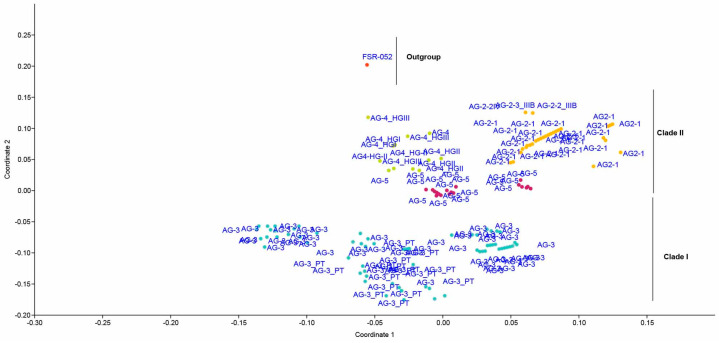
Principal coordinate analyses (PCoA) ordination of *R. solani* anastomosis groups. (AG) based on a Gower distance of sequence similarity matrix.

**Figure 8:**
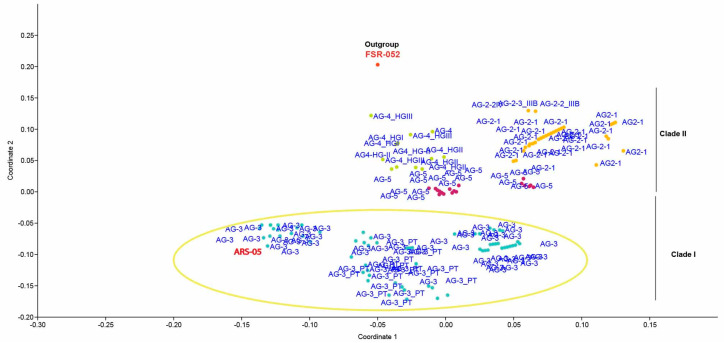
Principal coordinate analyses (PCoA) ordination of *R. solani* anastomosis groups. (AG) based on a Gower distance of sequence similarity matrix. Isolate ARS-05 makes a cluster with AG-3, which indicates isolates associated with black scurf are mostly AG-3.

The phylogenetic trees derived from Maximum Likelihood (ML), Maximum Parsimony (MP), and Neighbor-joining (NJ) analyses revealed that AGs or AG subgroups were closely grouped together, regardless of their origin. The results presented in the table depict the percentages of sequence identities within and between different clades and subclades of the rDNA-ITS sequences of *R. solani* AG. This genetic analysis illuminates the relationships and genetic diversity among the various clades and subclades, offering insights into the evolution and dissemination of the pathogen. In Clade I, the sequence identity within AG-3 is 96.5–97%, showcasing a relatively high genetic similarity among isolates belonging to this subgroup. For the 3PT subclade, the sequence identity ranges from 84% to 93.3%. AG-2 exhibits a sequence identity of 89.1–100%, demonstrating a broad genetic range within this clade. Clade II encompasses multiple subclades, each displaying distinct sequence identities. AG-2-1 and AG-2-2 exhibit sequence identities of 65–90% and 82.6–94%, respectively. AG-4 demonstrates remarkable genetic similarity with a sequence identity of 98.4–100%. AG-5 displays a wider range of sequence identities, spanning from 57% to 90.3%, indicating significant genetic diversity within these clades (Table 2, Figure 7 and Figure 8).

## 3. Discussion

Soil-borne pathogen *R. solani* has the potential to inflict severe crop losses and economic devastation in potato-growing regions across the world [28]. All the mentioned AG groups related to potatoes discussed in the present research belong to *R. solani* and the prevalence of AGs differed widely geographically. Most of the AGs were recorded from infected potato plants in New Zealand and China [8]. The discovery of new anastomosis groups (AGs) associated with potatoes in New Zealand suggests that *R. solani’s* host range and genetic diversity are increasing [30]. AG-3PT was discovered to prevail on potatoes with black scurf in New Zealand, which is consistent with prior investigations conducted in other regions of the world, while AG-2-1 was only discovered in a few places [30]. A significant quantity of losses related to *R. solani* AG-2Nt isolates, on the other hand, suggests that this subgroup also poses a major danger to potato quality and production [31]. The reports of a few AGs from Sweden, Namibia, Mauritius, Switzerland, South Africa, the United States, Japan, Australia, the United Kingdom, Finland, France, Spain, Switzerland, and Jordan in this study were most likely due to insufficient collection or purification techniques [32]. Black scurf and stem canker, which are mostly caused by the AG-3 group, are responsible for 30% of yield losses worldwide [33]. The fungus *R. solani* causes significant economic losses for potato growers in Sweden, particularly in locations with intense potato crop rotations [34]. From potato crops in northern China, the following anastomosis groups have been extracted: AG-1-IB, AG-2-1, AG-3, AG-4 (comprising the three homogeneous groups: HGI, HGII, and HGIII), AG-5, and AG-11 [35]. In Finland, 15–20% yield losses have been reported on potatoes due to *R. solani* per annum [36].

In our study, AG-3 was found to be a widely distributed anastomosis group on potatoes, as almost every country reported this group as mentioned above in this study, and the results of some other studies reported the same ideas about the prevalence of AG-3 on potatoes. In China, AG-3 PT of *R. solani* was the most prevalent fungus-producing black scurf and stem canker on potatoes [37]. Similarly, the authors of the reference [38] discovered that AG3 caused 94% of black scurf infections, with AG2-1 accounting for 4%, and AG5 accounting for 2% of samples. The authors of reference [39] found that 96% of black scurf was caused by AG3, whereas AG2-1 accounted for 4%. According to the findings of a study conducted in the United Kingdom, most of the isolates (92%) belonged to AG3PT, while others (67%) belonged to AG2-1. AG5 was represented by only one recovered isolate (0.7%) [40]. AG-4 and AG-5 are more prevalent in warmer environments and have been documented as primarily causing canker symptoms on potato plants, with minimal or no contribution to the formation of black scurf on tubers [8]. Moreover, in the context of Mexico, AG-4 was solely detected during the flowering period of potato growth, whereas AG-3 was observed on plants across all growth stages [27]. AG-3 PT was found to be predominant in Northern Australia [41]. The frequency with which AGs are reported is not indicative of whether they are especially dangerous on potatoes, e.g., several AG-2-1 samples caused serious damage to the potatoes and malformed tubers, while others resulted in moderate lesions or were not pathogenic [8]. As a result, AG diversity, frequency, and distribution may be influenced by host–pathogen association conditions, genetic versatility, and level of adaptation [32]. Host–pathogen associations, genetic versatility, and level of adaptation may influence the diversity, frequency, and distribution of anastomosis groups (AGs), whereas root-associated microbial populations have been shown to regulate the dispersion of AGs [42].

The precise examination of the rDNA ITS1-5.8S-ITS2 region through molecular characterization stands as the most precise approach for studying the phylogenetics and genetic diversity of *R. solani’s* AGs and AG subgroups [43]. By utilizing sequences from the rDNA ITS1-5.8S-ITS2 region sourced from the NCBI GenBank, it was managed to infer the phylogenetic relationships within AGs and AG subgroups. The employment of MP, NJ, and ML analysis for phylogenetic exploration revealed the division of AG into two distinct clades in this current investigation. Clade I encompassed AG-3 isolates, while Clade II contained isolates of AG-2, AG-4, and AG-5. This second clade was further fragmented into three separate subclades, each supported by reliable bootstrap values. Within these subclades, the AG-2 subgroup was represented by one subclade housing both AG-2-2 and AG-2-1 isolates, whereas the remaining two subclades encompassed AG-4 and AG-5 isolates. Our study showed that among the ten isolates (ARS-01-ARS-10), collected from potato fields of Multan, Pakistan, ARS-5 showed severe black scurf severity on the Kuroda variety of potato. The morphological characters showed that these ten isolates belonged to *R. solani* and AG-3 anastomosis groups. PCoA analysis revealed that the severe isolate ARS-05 clustered with AG-3 of the worldwide isolates. Previous studies have consistently demonstrated the predominance of AG-3 in association with black scurf disease in potatoes [32]. According to the authors of the reference [44], most isolates of *R. solani* infecting potatoes belong to AG-3, as determined by molecular characterization of the internal transcribed spacer (ITS) 1-5.8S-ITS2 rDNA region. Proteomic analysis of *R. solani* identifies infection-specific, redox-associated proteins and insight into adaptation to different plant hosts [44]. An unidentified binucleate Rhizoctonia isolate was obtained from a stem canker lesion, causing mild lesions on potato sprouts. Binucleate isolates of Rhizoctonia have been observed to display mild or non-pathogenic characteristics in soil within potato fields [45]. Some of these isolates may induce stem canker lesions or the telomorphic stage on the stem, as observed in various studies [32,45,46]. Potatoes in Pakistan are infected predominantly by AG-3 anastomosis groups of *R. solani*, as our results are in line with the authors of the reference [47,48], where a survey across eight agro-ecological zones revealed varying levels of black scurf and stem canker prevalence, with zone two (Punjab province) exhibiting the highest disease prevalence, incidence, and severity, while zone seven (northern Pakistan) reported lower levels of infection. In addition to Pakistan, black scurf and stem canker are widespread in Bangladesh and India. A survey conducted in major potato-growing areas of Bangladesh between 1999 and 2000 and 2000 and 2001 recorded disease incidences ranging from 1.84% to 23.99% [49,50,51]. In India, black scurf and stem canker persist in regions with continuous potato cultivation, with disease intensity varying by location. Areas with consecutive potato cultivation, such as Punjab, Haryana, Rajasthan, Himachal Pradesh, Uttar Pradesh, Bihar, etc., face an elevated risk of spreading diseases. The impact includes a 25% decrease in harvest in hilly regions and a 10% decrease in plain regions [49,50,51]. Estimates of production losses in Nepal attributed to black scurf and stem canker range from 5% to 60%, contingent upon the seasonal weather conditions and the crop rotation practices employed [52]. In Saudia Arabia, black scurf and stem canker were widespread and the most destructive diseases [53]. Black scurf and stem canker were detected in 109 samples of potato seeds imported to Jordan, with a prevalence of 42.4%, an incidence of 2.5%, and a severity of 0.46% observed across all seed lots [5]. Potato diseases are widely believed to be spread primarily through the movement of potato seed roots across both domestic and international borders [54]. Black scurf and stem canker are widely distributed in different countries including the United States, South Africa, New Zealand, Pakistan, Finland, Turkey, Saudi Arabia, Egypt, and France [55].

Yields are projected to decline by 30–40% in temperate zones and 19.2–34% in tropical and subtropical regions due to black scurf and stem canker, with a corresponding reduction in market value and production by approximately 30%. Furthermore, the diseases significantly impacted sprouting (11% reduction), stem count (70% decrease), and overall production (68% reduction), while fields with susceptible cultivars experienced elevated post-emergence wilting (27.2%), necrotic stem count (98.8%), and aerial tuberization (14.7%) [56,57].

In various geographical regions worldwide where potatoes are cultivated, distinct anastomosis groups (AGs) of *R. solani* were identified. *R. solani* isolates were gathered from various potato-growing zones in Pakistan, revealing that the occurrence percentages of AG3, AG5, and AG4 were 81.89%, 8.66%, and 5.51%, respectively. These findings underscored the predominance of AG3 compared to other groups [42]. Initially, AG-3 was classified as a single disease population exclusively affecting potatoes, yet AG-3 has now been shown to infect aubergines, tomatoes, and tobacco as well [54]. Cultural characteristics, fatty acid composition, and pathogenicity have all helped scientists distinguish AG3 strains from potatoes and tobacco [58]. Subsequently, AG3 was subdivided into AG-3PT (potato type), AG3TB (tobacco type), and AG3TM (tomato type), distinguished by differences in their nuclear ribosomal DNA ITS genomes. Additionally, while other anastomosis groups (AG2, AG4, AG5, AG7, AG8, and AG9) are associated with potato diseases worldwide, they generally exhibit lower levels of destructiveness compared to AG-3 [59]. AG3 has two subgroups, and AG3-PT is one of them, identified as the most widespread AG worldwide, infecting all underground components of potato plants at any stage of development and linked with potato diseases, while AG3-TB, on the other hand, is generally linked with tobacco [59]. These two subgroups are genetically different and have different morphological appearances, fatty acid profiles, and pathogenicity [59]. However, AG4 was the most common AG linked to stem lesions in the warm plains of Peru. Other AGs cause disease symptoms on potato stems, stolons, roots, and tubers in several other regions around the world. The widespread presence of AG3 may represent the disease’s population dynamics, likely due to international tuber trade. Cropping practices also influence the presence of AGs in a particular region [60]. Typically, isolates from black scurf on potatoes or other plant sections are AG3, while soil samples from potato fields may contain a wider diversity of AGs. On the surface of the tuber, deformations, and corky or scabby sores often harbor isolates of AG5, AG3, and AG2-1. Different AGs were found in diseased potato plants collected from areas with previous potato cultivation and those without [61]. The determination of AG linked to potatoes is influenced by agricultural practices, as indicated by the correlation between the frequency of various AG and different farming methods and cultural practices. Hence, the population organization of *R. solani* may be significantly influenced by cropping history and cultural practices. The ability of AG3 to generate more sclerotia than other potato-implicated AGs is a crucial factor that may explain the predominance of AG3 in potatoes [62].

The disparity in AG prevalence across regions may be attributed to a multitude of factors, including climate conditions, cropping practices, and genetic interactions between the pathogen and host. The emergence of AG-3PT as a prevalent pathogen in potato production regions raises concerns about its potential impact on yield and quality. Similarly, the persistence of AG-2Nt isolates in certain regions demands vigilant monitoring due to their substantial threat to potato crops [62]. The incorporation of modern agricultural techniques, including precision agriculture and synthetic biology, offers innovative avenues for mitigating the impact of *R. solani* [63]. Precision microbiome engineering and synthetic biology offer the potential to enhance soil health and strengthen plants’ defense mechanisms against pathogens [64]. Machine learning models based on comprehensive data sets contribute to disease prediction and management, enabling timely interventions.

## 4. Materials and Methods

### 4.1. Sample Collection, Fungal Isolation and Identification

About 100 tubers of potato cv Kuroda displaying characteristic symptoms of *R. solani*, including black scurf were collected from potato fields of Multan, Pakistan from March to June 2023. The collected samples were transported to the laboratory of plant pathology at the Department of Plant Pathology, Bahauddin Zakariya University, Multan. Here, efforts were made to isolate the pathogen and scrutinize its phenotypic and pathogenic characteristics. To ensure effective separation of *Rhizoctonia*, infected potato tubers displaying evident disease symptoms underwent a thorough rinse in running water and were subsequently dried for four hours in a laminar flow cabinet. Small sections of infected tissue, measuring 4 mm in diameter and 5 mm in depth, were precisely excised using a sterile scalpel blade. These sections were then plated onto petri plates containing 1.5% water agar (WA) supplemented with streptomycin sulfate (Sigma-Aldrich, St. Louis, MO, USA) at a concentration of 50 mg/liter. The plated samples underwent an incubation period of 48 h at 28 ℃. Upon completion of the incubation, colonies of each isolate identified as *Rhizoctonia* spp., which were subjected to microscopic examination, and fungal hyphal tips were carefully collected and subsequently placed on potato dextrose agar (PDA; Biolab, Budapest, Hungary) and were incubated for three days at 28 °C. Pure cultures of *R. solani* isolates were initially identified through the visual observation of macromorphological characteristics using the naked eye. The identification was subsequently confirmed under the microscope following the criteria outlined by the authors of the reference [65]. The identified fungi were then sub-cultured on PDA slants and stored at 5 °C for subsequent studies. The anastomosis groups were determined according to the methods mentioned in the previous studies [65,66].

### 4.2. Pathogenicity Test

To assess pathogenicity, 10 isolates of *R. solani* were cultivated on sterilized sorghum grains in 500 mL conical flasks for a duration of five days. Forty certified pathogen-free seed tubers (cv. Kuroda) were planted in 3 L greenhouse earthen pots (three tubers for one *R. solani* isolate in four earthen pots along with control as three replications in a completely randomized design) and subjected to inoculation with 10 g of sorghum grains colonized by *R. solani*. Control tubers were inoculated with sterile sorghum grains only. Following inoculation, the plants were maintained in a greenhouse under a 12 h photoperiod at a temperature of 27 ± 2 °C. Approximately 60 days after inoculation, symptoms resembling black scurf, consistent with those observed on potato tubers harvested from the field, were evident on tubers from the plants that had been inoculated. In contrast, no symptoms were noted on tubers from the control plants. The severity of the black scurf was assessed on a scale ranging from 0 to 4, following the criteria established by the authors of the reference [67], 50 days after the initial sowing. This grading was determined by evaluating the percentage of the tuber surface displaying symptoms of the disease.

### 4.3. Molecular Identification

The DNA was extracted from 50 mg of mycelial growth of one of the severe isolates. AGR-05 using the Qiagen Kit, was dissolved in 100 µL of elution buffer. To assess the concentration and purity of the obtained DNA, a Genomic DNA preparation kit was employed. The concentration of the DNA sample was adjusted to 6 ng/µL using TE buffer (pH 8.0), while the purity levels reached 90–97% with ratios between 1.7 and 1. The fungus’s identity was verified through the sequencing of a section of the internal transcribed spacer (ITS) region within ribosomal DNA (rDNA). This was achieved using the ITS1-F and ITS4 primers as described by the authors of [68]. The amplification protocol for PCR involves a series of steps. Initially, a denaturation phase at 94 °C for 2 min was conducted in a single cycle. Subsequently, 35 cycles were carried out, each consisting of denaturation at 94 °C for 30 s, annealing at 52 °C for 30 s, and extension at 72 °C for 3 min. A final extension step at 72 °C for 10 min concluded the amplification process in a single cycle. The resulting sequences were then subjected to analysis using BLAST^®^ [69], which involved comparing the sequences against the NCBI sequence database (National Center for Biotechnology Information, GenBank) at www.ncbi.nlm.nih.gov/genbank/ accessed on different dates of July 2023. This analysis aimed to identify sequences like those with known AGs. While providing valuable insights, this study has certain limitations. The reliance on GenBank data might introduce biases and undersampling, influencing AG representation. Nonetheless, this research’s implications are substantial.

### 4.4. Data Collection for Assessment of Anastomosis Groups (AGs) Associated with Potato

For the assessment of anastomosis groups, the research articles published in peer-reviewed journals from January 2001 to July 2023 were collected. Articles had to fulfill specific requirements to be part of the research, such as stating the GenBank accession codes and sequence details regarding anastomosis groups (AG), geographical background, strains, as well as AGs that produce signs and symptoms on potatoes. Additionally, the articles needed to describe extraction/recovery of fungus from different sources, including infected tubers, stems of potato crops, and surrounding soil (e.g., rhizosphere soil, topsoil) of infected potato plants showing black scurf. For additional study, the acquired data on AG, strains, geographic background, and isolation methods were assembled. The relative occurrence of each AG concerning other AGs was quantified using a modified formula for this specific study. The number of studies conducted on each AG or AG subgroup found in the scientific journals (n) was expressed as a percentage of the total number of studies involving all AG/AG subgroups (N). This calculation was represented as F = 100 × (n/N). AGs with higher percentages of studies were identified as the most frequently reported or extensively studied AGs associated with potato crops worldwide.

### 4.5. Phylogenetic Analysis

Before conducting a phylogenetic analysis on the aligned sequences, the most appropriate substitution model was determined using the jModelTest version 2.1.6 software package. This selection was guided by the Akaike Information Criterion (AIC) and Bayesian Information Criterion (BIC) estimates. The Tamura–Nei model was ultimately chosen to construct the phylogenetic tree. The phylogenetic trees were created using three methods: Maximum Likelihood (ML), Neighbor-joining (NJ), and Maximum Parsimony (MP). To assess the strength of each phylogenetic tree, bootstrapping was performed with 1000 random samples.

### 4.6. Principal Coordinate Analysis (PCoA) and Sequence Similarities

The software tool MatGAT version 2.0 was employed to calculate the genetic similarity percentages among isolates within AGs and AG subgroups, as well as between distinct AGs and AG subgroups. To visualize the results, principal coordinate analyses (PCoA) were conducted on the matrices of paired sequence similarities. These analyses were carried out using the Gower similarity index and implemented with the Paleontological Statistics software program for teaching and data assessment (PAST) version 4.03.

## 5. Conclusions

This comprehensive study delved into the distribution and genetic diversity of *Rhizoctonia solani* anastomosis groups (AGs) associated with potato crops across various geographic regions. The findings unveiled a complex landscape of AG prevalence, highlighting AG-3 as the most widespread group, followed by AG-5 and AG-2-1. The prevalence of specific AGs varied significantly among different countries, with AG-3 being reported extensively in Sweden, China, and the USA, while AG-5 was prominent in Sweden and the USA. Furthermore, our study showed that ARS-05 was most severe on potatoes. Anastomosis grouping indicates ARS-05 belonged to AG-3 of *R. solani*. This indicates that potatoes in Pakistan are infected by AG-3. The phylogenetic analysis demonstrated distinct clades and subclades, revealing both shared genetic ancestry and geographic divergence among AGs. Despite the valuable insights gained from this research, several limitations merit consideration. Firstly, the reliance on data available in GenBank may introduce biases in AG representation and geographic coverage. Additionally, the absence of certain AGs in specific regions could be attributed to undersampling or underreporting, potentially skewing the prevalence analysis. Furthermore, the lack of genetic information from some countries may limit the accuracy of the geographic distribution analysis. To build upon these findings, future research endeavors should strive to address the limitations of this study and explore additional dimensions of AG diversity and pathogenicity. Expanding the scope of data collection through targeted field surveys and metagenomic analyses would enhance the accuracy and comprehensiveness of AG prevalence assessments. Moreover, incorporating advanced sequencing techniques, such as next-generation sequencing, could provide a more detailed understanding of genetic variations within AGs. In terms of practical applications, the insights gained from this study lay the groundwork for developing region-specific management strategies to mitigate the impact of *R. solani* on potato crops. Efforts should be directed toward harnessing the power of precision agriculture, data-driven disease prediction models, and genetic engineering to enhance crop resilience and reduce losses caused by AGs. Additionally, collaborative initiatives between researchers, agronomists, and policymakers can facilitate the implementation of sustainable practices and innovative technologies to safeguard potato cultivation in the face of evolving AG dynamics. In conclusion, this research offers a significant contribution to the understanding of *R. solani* AGs’ distribution and genetic diversity in potato crops worldwide. By unveiling the intricate relationships among AGs, geographic origins, and phylogenetic relatedness, this study provides a stepping stone for future studies to refine disease management strategies and foster sustainable potato production systems.

## Figures and Tables

**Table 1:** The number of studies showing AGs associated with potatoes in different geographical origins.

Anastomosis Groups	Subgroups	Sweden	Namibia	Mauritius	Switzerland	China	South Africa	USA	Japan	New Zealand	Finland	France	UK	Spain	Australia	Jordan	Total
AG-2	AG-2-1	2	----	----	----	2	----	3	----	24	2	1	3	----	1	----	38
----	AG-2-2IIIB	----	----	----	----	----	----	2	----	----	----	----	----	----	----	----	2
----	AG-2-2IV	----	----	----	----	1	----	----	----	----	----	----	----	----	----	----	1
AG-3	3	13	2	1	2	----	3	----	----	116	1	6	----	2	----	20	166
----	AG-3 PT	----	----	----	2	42	----	----	----	----	----	----	----	----	----	----	44
AG-4	4	----	----	----	----	----	----	----	----	1	----	----	----	----	----	----	1
----	AG-4 HGI	----	----	----	----	12	----	----	----	----	----	----	----	----	----	----	12
----	AG-4 HGII	1	----	----	----	5	----	1	1	----	----	----	----	----	----	----	8
----	AG-4 HGIII	----	----	----	----	----	3	----	----	----	----	----	----	----	----	----	3
AG-5	----	1	----	----	----	13	----	----	----	1	----	----	----	----	----	----	15
Total	----	17	2	1	4	75	6	6	1	142	3	7	3	2	----	20	290

**Table 2:** Percentages of sequence identities of rDNA-ITS sequences of *R. solani* AG within and between clades and subclades.

			Clade I		Clade II							
			AG-3		AG-2		AG-4				AG-5	
	Clades		3	3PT	AG-2-1	AG-2-2	4	AG-4-I	AG-4-II	AG-4-III	5	Outgroup
Clade I	AG-3	3	96.5–97									
		3PT	84–93.3	89.1–100								
Clade II	AG-2	AG-2-1	65–90	82.6–94	98.4–100							
		AG-2-2	57.8–84	79.8–85.5	83.6–86	97–100						
	AG-4	4	62.6–86	74.8–88.6	87.9–88	81.3–82.8	97–100					
		AG-4-I	62.2–87.1	79.2–87.8	80–88	79.7–83.1	87–95.2	84–100				
		AG-4-II	74–85.6	81.1–86.6	80–88	79.8–81.9	87.2–94	96.7–97	100			
		AG-4-III	62–85	68–82.2	82.8–88.5	75.3–83.6	86.1–91.3	75–92.7	72–90	93–100		
	AG-5	5	57–90.3	83.7–90.5	84.8–87.7	82.3–85.5	81.8–83.3	82.6–84.1	86.8–87.8	76.3–84.3	97.8–100	
Outgroup			57.1–68.6	63.7–69	67–68	67.4–68.5	67.9–68	67.3–67.7	67.4–67.9	62–67	65.2–66.3	100 *

* Range could not be calculated for one isolate having one sequence.

## Data Availability

Data are contained within the article.
